# Molecular Mechanisms Involved in Intrarenal Renin-Angiotensin and Alternative Pathways in Diabetic Nephropathy - A Review

**DOI:** 10.1900/RDS.2021.17.1

**Published:** 2021-08-01

**Authors:** Elham Bahreini, Yousef Rezaei-Chianeh, Mohsen Nabi-Afjadi

**Affiliations:** 1 Department of Biochemistry, Faculty of Medicine, Iran University of Medical Sciences, Tehran, Iran,; 2 Department of Biochemistry, Faculty of Biological Science, Tarbiat Modares University, Tehran, Iran.

**Keywords:** nephropathy, vascular endothelial growth factor, glomerular filtration rate, angiotensin, angiotensin-converting enzyme, local renin-angiotensin system

## Abstract

Uncontrolled or chronic hyperglycemia causes kidney failure induced by the dysfunction of biomolecules and upregulation of inflammatory cytokines and growth factors. The reninangiotensin system (RAS) is incorporated in the regulation of renal hemodynamics. In a healthy state, local RAS is independent of systemic RAS. However, in pathological conditions such as chronic hyperglycemia, angiotensin II (Ang II) increases locally and causes tissue damage, mainly through the induction of oxidative stress, inflammation, and upregulation of some growth factors and their receptors. Such tissue events may cause disruption of the glomerular filtration barrier, thickening and hypertrophy of the glomerular basement membrane, microvascular hyperpermeability, proteinuria, and finally decrease in the glomerular filtration rate (GFR). Reduced GFR causes the kidney to sense falsely a low blood pressure condition and respond to it by stimulating systemic and local RAS. Therefore, patients with diabetic nephropathy (DN) suffer from chronic hypertension. In contrast to local RAS, there are alternative pathways in the kidney that act protectively by reducing tissue Ang II. Such autoregulatory and protective mechanisms are weakened in chronic kidney disease. Previously, it was presumed that systemic RAS inhibitors such as ACE inhibitors (ACEIs) or angiotensin receptor blockers (ARBs) could prevent renal damage by controlling blood pressure and proteinuria. However, the progression of renal failure to end-stage renal disease (ESRD), despite such treatments, indicates the presence of factors other than Ang II. This review highlights the molecular mechanism in renal disease and discusses pharmaceutical and therapeutic approaches.

## 1 Introduction

Diabetic nephropathy (DN) is a long-term kidney disease that occurs in people suffering from chronic diabetes mellitus [1]. As DN progresses, the most important structural changes occur in glomeruli. Glomerulonephritis (GN) is an inflammatory process in the glomeruli or small blood vessels in the kidney that is characterized by DN through diffuse or nodular sclerosing glomerulonephritis [2]. Glomerulosclerosis is associated with the thickening of the glomerular basement membrane (GBM) induced by an increase in the mesangial matrix that contains large amounts of collagen, especially types IV and VI, laminin, and fibronectin [3]. Mesangial expansion restricts the glomerular capillary surface area, disturbs the filtering process, and allows the protein leaking from the blood into the urine [4]. Thus, the early stages of glomerulosclerosis are defined by proteinuria that is characterized by a positive microalbuminuria test caused by the progression of glomerular hypertrophy, GBM thickening, hyperperfusion, and hyperfiltration [5].

## 2 Uncontrolled hyperglycemia promotes vascular endothelial growth factor (VEGF) in the kidney

**Figure 1.**
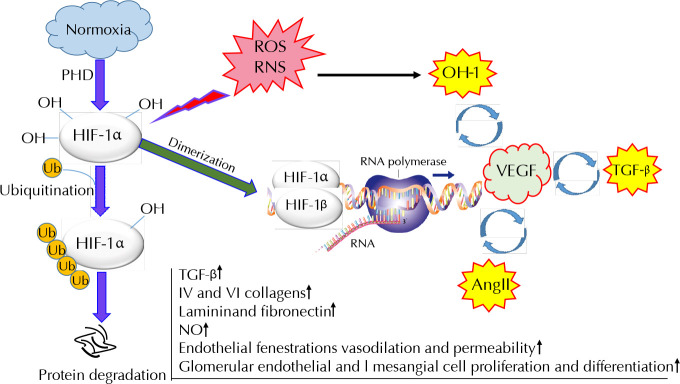
**Degradation of HIF-1α in normoxia, its dimerization with HIF-1β in oxidative conditions, and its adaption of the role of a transcription factor**. Induced VEGF stimulates the expression of TGF-β, IV and VI collagens, laminin, and fibronectin, increases NO production, endothelial fenestration, vasodilation, and permeability, and induces glomerular endothelial and mesangial cell proliferation and differentiation. *Abbreviations:* Ang II - angiotensin II; NO - nitric oxide; PHD - prolyl hydroxylase; HO-1 - hemoxygenase-1; RNS - reactive nitrogen species; ROS – reactive oxygen species; TGF-β - transcription growth factor; VEGF - vascular endothelial growth factor.

The abnormalities in the diabetic kidney begin with long-term, poorly controlled hyperglycemia. Uncontrolled or chronic hyperglycemia leads to the non-enzymatic formation of advanced glycation end products (AGEs) through a variety of pathways, including the formation of a Schiff base, which alters the charge, solubility, and conformation of proteins [6]. AGE modification of proteins may result in abnormal molecular function, interaction, or signaling which contribute to renal expression of inflammatory cytokines and growth factors, like interleukin 1 (IL-1), interleukin 18 (IL-18), interferon gamma (IFN-γ), tumor necrosis factor alpha (TNF-α), transforming growth factor β (TGF-β), and insulin-like growth factor 1 (IGF-1) [7]. The production of reactive oxygen species (ROS) and oxidative stress coexist with inflammation and intensify mutual generation [8]. Releasing such factors from intrinsic renal cells or blood-borne cells plays an important role in renal fibrosis and mesangial expansion by the accumulation of extracellular matrix and interstitial proteins, especially collagen [9]. Increased ROS production and a shift towards fatty acid utilization enhance renal oxygen utilization and tissue hypoxia [10]. Hypoxia and oxidative stress conditions inhibit hypoxia-inducible factor 1α (HIF-1α) from degradation and allow HIF-1α to be bonded to HIF-1β (Figure 1) [11]. The stabilized heterodimer activates transcription of several genes involved in angiogenesis, cell proliferation, and anaerobic metabolism needed for adaptation in hypoxia and ischemia. Growth factor signaling pathways also upregulate HIF-1α levels via oxygen-independent mechanisms [12].

Among several angiogenic factors induced by HIFs, VEGF plays a critical role in minimizing hypoxia-induced events through neovascularization [13]. It is more intensely secreted from podocytes and the thick ascending limb and to a much lesser extent from the proximal and distal tubules [14]. However, VEGF receptors are mainly limited to endothelial cells in the glomerular capillary loops and peritubular capillaries [14, 15]. VEGF, along with other stimulators, including angiopoietins (ANGPTs), epidermal growth factor (EGF), semaphorin 3A (SEMA3A), TGF-β, and C-X-C motif chemokine ligand 12 (CXCL12), signal in paracrine ways and regulate glomerular filtration barrier (GFB) function [16]. During the early stage of DN, VEGF levels and its receptors are upregulated in the glomerulus.

Several studies indicate that VEGF expression may be upregulated through various mechanisms. Heme oxygenase 1 (HO-1), overexpressed by ROS and nitric oxide (NO), enhances VEGF synthesis and is involved in angiogenesis [16]. VEGF also regulates HO-1 expression and activity through a positive-feedback loop [17]. In addition to sodium retention, aldosterone upregulates the expression of VEGF in the epithelial cells of the cortical collecting duct and stimulates inflammation and fibrosis by ROS generation and interaction with RAS [18]. Ang II has previously been reported to reinforce VEGF-mediated angiogenesis via the upregulation of VEGF receptors [19-21]. Ang II stimulates VEGF expression mainly through angiotensin II receptor 1 (AT1R) and increases the expression of VEGF-R2 mostly through angiotensin II receptor 2 (AT2R) [22]. Excess VEGF results in stimulating TGF-β expression, enhancing IV and VI collagens, laminin and fibronectin synthesis, augmenting NO production, increasing endothelial fenestrations, vasodilation, and permeability, and inducing glomerular endothelial and mesangial cell proliferation and differentiation. These events result in the disruption of GFB, thickening and hypertrophy of GBM, microvascular hyperpermeability, proteinuria, and finally decrease in glomerular filtration rate (GFR) [15, 23].

## 3 Reduced GFR and constant hypertension in diabetes

The juxtaglomerular cells localized mainly in the walls of the afferent arterioles, and to a lesser extent in the wall of the efferent arterioles, release renin in response to a drop in blood pressure or when biochemically stimulated by macula densa cells [24]. The macula densa is an area at the point between the thick ascending limb of the Loop of Henle and the distal convoluted tubule. It consists of specialized cells, which are salt sensors and stimulate the juxtaglomerular apparatus by generating paracrine chemical signals [25]. A reduction in GFR during glomerulonephritis results in a decrease in the volume of filtered fluid and a reduction in the concentration of sodium reaching the macula densa [26]; both stimulate juxtaglomerular cells to release renin into the bloodstream. Following the response to blood renin by circulating RAS, blood pressure rises, i.e. the blood pressure regulatory system in the kidney falsely senses a low blood pressure condition and responds to it. Therefore, patients with diabetic nephropathy suffer from constant hypertension. Generally, proteinuria, increased blood pressure, and decreased GFR are the clinical manifestations of diabetic nephropathy, which gradually progresses to end-stage renal disease (ESRD) [27].

## 4 Angiotensin II in systemic and local renal RAS

Renin hydrolyzes blood angiotensinogen to form Ang I which then is converted to Ang II by angiotensin-converting enzyme (ACE) present in many tissues, particularly in the pulmonary vascular endothelium [28]. Ang II increases blood pressure through acting on arteriolar smooth muscle to cause vascular constriction, on the zona glomerulosa of the adrenal cortex to stimulate aldosterone secretion and sodium reabsorption from the kidney, on the posterior pituitary gland to release vasopressin which elevates water resorption in the kidney, and on sympathetic fibers to release norepinephrine [29, 30]. In addition to systemic RAS, Ang II is also produced in local renal RAS [31, 32]. As is well known in both normal and pathological conditions, the role of local RAS in regulating renal hemodynamics is independent of that of systemic RAS. Besides tubularglomerular feedback and myogenic responses to renal autoregulation, local Ang II also affects the tonicity of both afferent and efferent arterioles, which raises the systemic arterial blood pressure and decreases renal blood flow [33]. Despite this drop in renal blood flow, the glomerular pressure should be maintained to continue blood filtration and maintain GFR [34].

Although Ang II constricts both afferent and efferent arterioles, the resistance of efferent arterioles is greater than that of afferent arterioles because of their smaller diameter. Since AT2R is more abundantly present in the afferent arterioles than AT1R and because of the release of NO, the constriction of the afferent arterioles is stronger than that of the efferent arterioles [26]. These different tonicities in two vascular compartments force the blood to accumulate gradually in the glomerulus and increase the glomerular pressure. Also, Ang II can reduce the surface area available for filtration via elevating prostaglandin levels in glomeruli and constricting the glomerular mesangium [35]. This results in the maintenance of normal blood filtration by an increase in filtration fraction (i.e. the ratio of GFR to renal plasma flow). Following the stimulation of water and sodium reabsorption in the proximal tubule by local Ang II, the feedback mechanism is activated in macula densa, which reinforces the process. This plays an important role in the progression of hypertension and renal injury [35].

**Figure 2.**
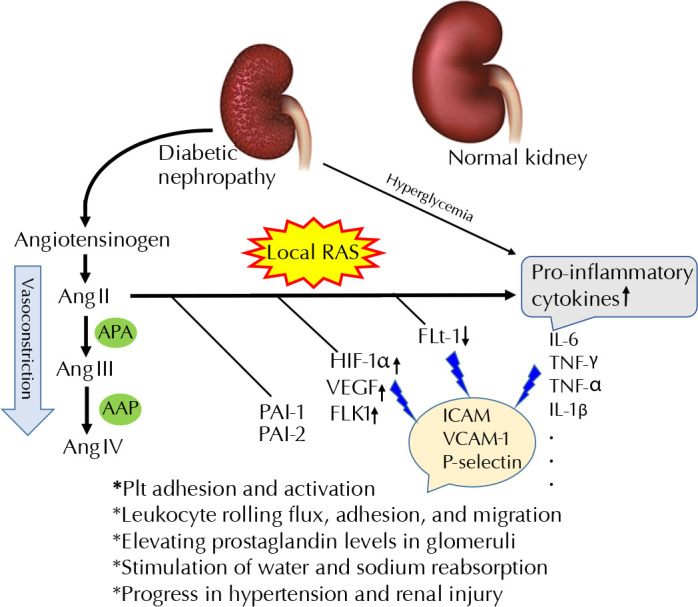
**Induction of local Ang II by the activation of local RAS which results in hypertension and endothelial and arterial injuries**. Ang II induces the stimulation of platelet adhesion and activation, leukocyte rolling flux, adhesion, and migration. It also causes the increase in prostaglandin levels in glomeruli, reabsorption of water and sodium, and progress in hypertension and renal injury. Conversion of Ang II to Ang III and then Ang IV by APA and AAP, respectively, reduces the destructive properties of Ang II and vascular contraction. *Abbreviations:* APA - aminopeptidase A; AAP - alanyl amino-peptidase; Flk-1 - fetal liver kinase 1 (also known as vascular endothelial growth factor receptor 2 (VEGFR-2)); Flt-1 - Fms-like tyrosine kinase 1 (also known as vascular endothelial growth factor receptor 1 (VEGFR-1)); HIF-1α - hypoxia-inducible factor 1 alpha; ICAM-1 - intercellular adhesion molecule 1; IFN-γ - interferon γ; IL-6 - interleukin 6; PAI - plasminogen activator inhibitor; Plt - platelet; RAS - renin-angiotensin system; TNF-α - tumor necrosis factor α; VCAM-1 - vascular cell adhesion molecule 1; VEGF -vascular endothelial growth factor.

As shown in Figure 2, Ang II induces platelet adhesion and releases plasminogen activator inhibitors (PAI-1 and PAI-2), the potent coagulant/atherogenic factors [36]. Ang II becomes converted to Ang III (Ang (2-8)) by aminopeptidase A (APA) generally located in RBCs and the vascular surface of most tissues, mainly in endothelial and mesangial cells of the glomerulus and brush border compartments of the kidneys with high activity [37]. Ang III has 40% of the vasoconstriction properties and 100% of the aldosterone-producing activity of Ang II [38]. Ang IV (Ang (3-8)) generated by alanyl aminopeptidase (AMP) or arginine aminopeptidase has lesser activity than Ang III [39].

## 5 Local RAS function in hyperglycemia

The hyperglycemic and inflammatory area of the diabetic kidney enhances the expression of local RAS components and the generation of local Ang II [40]. The autoregulatory mechanism of Ang II is disrupted in chronic kidney disease, which is due to renal hypertrophy and increased intraglomerular pressure. Increased renovascular hypertension activates the intratubular RAS in the entire nephron population. Such local RAS hyperactivity plays a serious role in renal tissue injury via Ang II [41]. According to the results of several animal studies, elevated Ang II, in turn, alters local levels of pro-inflammatory cytokines such as IL-6, IFN-γ, TNF-α, and IL-1β [42, 43], which are also stimulators of VEGF expression.

Liu *et al*. found an increase in the expression of VEGF, TGF-β, VEGFR2 (Flk-1), and their levels of phosphorylation in cultured podocytes stimulated with Ang II, with a smaller effect on VEGFR1 (Flt-1) [44]. Sanchez-Lopez *et al*. reported that VEGF gene expression and promoter activation increased in cultured tubuloepithelial cells treated with Ang II. They also observed that Ang II induced HIF-1α production and DNA-binding activity [45]. Ang II induces increased leukocyte rolling flux, adhesion, and migration via direct stimulation and augmentation of intercellular adhesion molecule 1 (ICAM1), vascular cell adhesion molecule 1 (VCAM-1), and P-selectin, independently of vasoconstrictor action (Figure 2) [42, 46]. This condition promotes the accumulation of macrophages in the kidney resulting in elevated local inflammatory cytokine levels and disease progression.

There are two main receptors for Ang II with opposite functions. The majority of biological actions and variety of pathophysiological effects of Ang II such as decreased renal blood flow, vasoconstriction, vascular smooth muscle cell proliferation, and renal tubular sodium reuptake are mediated by Ang II subtype 1 receptor (AT1R), while Ang II subtype 2 receptor (AT2R) counteracts the effects of AT1R and elicits protective effects via inhibiting cell proliferation and differentiation and promoting vasodilation and natriuresis [47]. Both receptors belong to G-protein-coupled receptors, but AT1R acts through Gq/11 [48] and subsequently phospholipase C and diacylglycerol. AT2R activates Gαs [49] and the cAMP-dependent pathway. In renal tissue, AT1Rs are distributed throughout the renal compartments and located predominantly in the glomerulus, afferent and efferent arterioles, and renal tubules [50]. AT2R is highly expressed in fetal kidney where it is involved in cellular differentiation and tissue development and rapidly decreases after birth [51]. AT2R is found, to a lesser extent than AT1R, in the vessels, glomeruli, and tubules of the adult kidney [52]. It is involved in natriuresis via the bradykinin/NO/ cGMP pathway that lowers blood pressure by increasing sodium excretion. However, the pathway is blocked by ACE via bradykinin degradation [53]. Interestingly, NO may have a positive feedback on AT2R upregulation [54].

Previous studies have suggested that Ang III is the regulating ligand in renal sodium excretion and mediates the AT2R-dependent natriuretic response [53, 55]. Although Ang III is the preferred AT2R agonist, both Ang II and Ang III stimulate vasodilation and decrease vascular resistance and natriuresis via activation of AT2R in the proximal tubules through the renal bradykinin/NO/cGMP signaling pathway [56]. However, studies have shown that Ang II must be converted to Ang III to induce AT2R-dependent responses [53, 57]. AT2R also lowers blood pressure by inhibiting renin synthesis and prevents atherosclerosis via inhibition of cellular proliferation and hypertrophy [58]. Generally, the characteristics attributed to AT2R include anti-inflammation, decreased sympathetic activity, anti-apoptosis, anti-fibrosis, and anti-growth [53, 59].

The AT4 receptor (AT4R), another angiotensin receptor, has been defined as the specific binding site for Ang IV. It has a general distribution in some tissues, including the kidney, adrenal gland, heart, and lung. AT4R is expressed in several renal cells with a mediatory role in the expression of PAI-1. However, its function in the kidney needs to be investigated [60]. The other local RAS components that counteract with Ang II signals are ACE2, Ang (1-7) and Mas receptors which are upregulated by AT2R.

## 6 Inhibition of RAS in diabetic nephropathy

The deterioration in kidney function may be significantly attenuated by effective control of blood pressure and reducing proteinuria [61]. As mentioned above, hyperglycemia activates local RAS by upregulating intracellular RAS components including renin, ACE, and chymase (an Ang II-generating enzyme) in mesangial cells, AT1R, AT2R, and renin in endothelial cells, and renin and AT1R in podocytes [62]. Some contradictory results have been reported on the effects of hyperglycemia on AT1R levels, but most of them have shown an increase in AT1R density [62, 63]. Hyperglycemia also increases angiotensinogen production in glomerular mesangial cells, podocytes, and endothelial cells. Ang II, in turn, stimulates the proximal tubule to express angiotensinogen and release it into the tubular fluid [62]. Therefore, urinary concentration of angiotensinogen may be a signal for kidney damage [64].

At first, it was presumed that RAS blockades such as ACE inhibitors (ACEIs) or that angiotensin receptor blockers (ARBs) could prevent renal damage by controlling blood pressure and proteinuria, but the progression of renal failure, despite such considerations, indicated the interference of factors other than Ang II [64]. In addition to the ACE pathway, hyperglycemia increases Ang II generation via non-ACE pathways, including chymase, kallikrein, cathepsin G, and elastase-2 responsible for Ang II formation in human tissues [65].

Chymase is a chymotrypsin-like serine protease which is mainly secreted by mast cells and to a lesser extent by cardiac fibroblasts and vascular endothelial cells and localized in tissue bound to heparin proteoglycans; such bonding protects the activity of chymase for several weeks [66]. Chymase is an important multifunctional enzyme in tissue remodeling and a potent pro-inflammatory agent with a role in converting Ang I to Ang II more effectively than ACE [67]. It is poorly expressed in glomeruli and other parts of the normal kidney, but significantly upregulated in diabetic kidneys because of inflammatory tissue conditions. Chymase promotes glomerulosclerosis and tubulointerstitial fibrosis via Ang II, and induces TGF-β formation directly from pro-TGF-β and indirectly through Ang II stimulation [66].

Kallikreins are a subgroup of serine proteases with various physiological functions, one type of which exists in the plasma with about fifteen closely related types in various tissues [67, 68]. Bradykinin and kallidin are the most important mediators in the kallikrein-kinin system and participate in blood pressure regulation as vasodilators. ACE possesses a strong affinity to degrade bradykinin. ACE inhibitors increase bradykinin levels in tissues which, in turn, cause mast cell degranulation and chymase release (tricking ACE inhibitors) [69, 70]. The kallikrein-kinin system, which is in counteraction with RAS, inhibits apoptosis, inflammation, and fibrosis via suppression of oxidative stress, TGF-β1 expression, and MAPK activation; it also has a protective effect against developing microalbuminuria [71, 72]. Therefore, independent of the vasodilatory effect, the system is capable of protecting the kidney from tubular damage, glomerulosclerosis, and perivascular remodeling. Clinical studies have shown that the excretion of kallikrein in the urine of diabetic patients with nephropathy is significantly lower than in diabetic patients without nephropathy and in healthy individuals [73].

Cathepsin G, a serine proteinase involved in the inflammatory responses, exists in high concentrations in the azurophil granules of neutrophils and peroxidase-positive granules of monocytes and macrophages and to lesser amounts in the blood stream and various tissues [74]. It was also capable of converting Ang I to Ang II and even of generating Ang II directly from angiotensinogen [75]. The half-life of free circulating cathepsin G is short, about 4-5 minutes, but binding to its native inhibitors, such as α1-antichymotrypsin in plasma or secretory leukocyte proteinase inhibitor in the kidney, as sources of active cathepsin G, increases its stability and can be reactivated by partial proteolysis of the inhibitor [76, 77]. However, little research has been done on the role of cathepsin G in kidney diseases until today.

Elastase-2, a serine protease elastase family member 2A, is widely found in several organs [78]. In addition to breaking down elastin and collagen, destroying bacteria by neutrophils, and playing a role in immunological balance, it participates in an alternative pathway for Ang II generation, especially in the presence of ACE inhibitors which increase elastase-2 contribution to Ang II generation [79].

## 7 Intrarenal RAS in diabetic nephropathy

**Figure 3.**
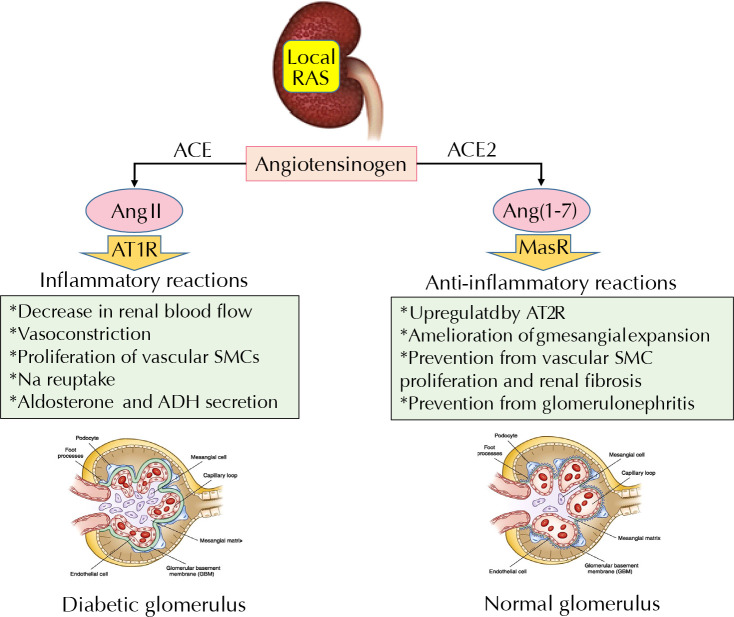
**Comparison of ACE and ACE2 and the function of their products**. The ACE pathway is an inflammatory process, and the ACE2 pathway, as an alternative pathway, is an anti-inflammatory process. The balance between the two pathways regulates glomerular function and renal homeostasis, which is disrupted in diabetic nephropathy. *Abbreviations:* ACE - angiotensin-converting enzyme; ADH - antidiuretic hormone; Ang (1-7) - angiotensin 1-7; Ang II - angiotensin II; AT1R and AT2R - angiotensin II receptor 1 and 2; MasR – Mas receptor (also known as G protein-coupled receptor); RAS - renin-angiotensin system; SMC - smooth muscle cell.

Diabetic nephropathy starts with a significant stimulation of juxtaglomerular cells to release renin [1, 20]. Both circulatory and tissue RAS appear to be overactivated in the development of glomerulosclerosis and renal fibrosis [4, 32]. Thus, early intervention via RAS inhibitors in diabetic patients with normal albuminuria seems rational. However, what are the natural pathways in the body that reduce or regulate the local RAS system? The components of the alternative RAS pathway including ACE2, Ang (1-7), and Mas receptors with proven cardio- and the reno-protective roles are upregulated by AT2R (Figure 3) [80]. ACE and ACE2 with 40-42% homology traverse the plasma membrane and also exist in soluble forms in the bloodstream when dissociated from the cell membrane [81]. Both co-localize in the brush border of mouse proximal tubules and different cell types of glomeruli [82]. In the glomerulus, ACE2 is present in epithelial and mesangial cells and ACE is mainly expressed in endothelial cells. In contrast to the fibrogenic, proinflammatory, and proliferative actions of the ACE/ Ang II/AT1 pathway, it has been demonstrated that the ACE2/Ang (1-7)/Mas axis has anti-inflammatory, anti-fibrogenic, and anti-proliferative properties and protects against glomerulosclerosis and Ang II-induced tubulointerstitial fibrosis [81, 82]. Activating Mas-related G-protein-coupled receptor antagonizes AT1R, reduces ROS and inflammation, and thus ameliorates renal injury. Animal studies have demonstrated a downregulation in kidney ACE2 in nephropathic kidney, possibly because of enhanced Ang II-mediated TGF-β/ Smad and NF-κB signaling pathways. Also, inhibition of ACE2 with an inhibitor like MLN-4760 accelerates albuminuria [81, 83]. Oudit *et al*. administrated recombinant ACE2 to mice treated with Ang II and observed an attenuation of increased blood pressure and a decrease in oxidative stress, renal Ang II levels, and fibronectin levels as a marker of fibrosis [84]. It can be concluded that ACE and ACE2 collaborate to regulate the intrinsic renal homeostasis by balancing the expression of Ang II and Ang (1-7).

Several small peptides have been recognized to originate from Ang II degradation with local physiological effects in the kidney, including Ang (1-7), Ang (2-8), Ang (3-8), Ang (3-4), Ang (1-5), and Ang (1-4) [85]. Among them, the protective role of Ang (1-7) in kidney complications, which is produced by ACE2 from the hydrolysis of Ang II and has an affinity for Mas receptor, has been studied in detail [86]. Although there are conflicting results for the effect of Ang (1-7) on the development of chronic kidney disease (CKD), most of them emphasize its protective role. According to several reports, the renoprotective effects of Ang (1-7) exerted by the molecular mechanisms include the inhibition of mechanisms induced by AGR [87] and the MAPk/ ERK1/2 pathway [88], a reduction in the expression of collagen IV, VEGF, TGF-β, NOX4, p47 phox, PKCb1, and PKCa, and the phosphorylation of Smad3 [88, 89]. Therefore, Ang (1-7) antagonize the effects of Ang II on tissue inflammation and fibrosis and on lipid accumulation by upregulation of the LDL receptor.

Although ACE2 is the main producer of Ang (1-7), there are other enzymes such as neprilysin (NEP) [90], prolyl-endo-peptidase (PEP) [91], and prolylcarboxy-peptidase (PRCP) [92] that can convert Ang I or Ang (1-9) to Ang (1-7). Among the aforementioned enzymes, NEP, a membrane-bound, zinc-dependent metalloendopeptidase (MME) with widespread tissue distribution has been abundantly detected in the kidney, mainly in the brush border of proximal tubular cells [90]. In addition to forming Ang (1-7) from Ang I cleavage, it degrades natriuretic and other vasoactive peptides including substance P, bradykinin, endothelin, and Ang II. Although some of the NEP activities like Ang (1-7) formation and Ang II and endothelin degradation help to prevent kidney failure, lowering the levels of natriuretics and other vasoactive peptides has unfavorable effects which exacerbate CKD [93]. Thus, targeting NEP may have potential benefits and adverse consequences. For example, NEP inhibition decreases blood pressure by increasing natriuresis and diuresis and by inhibiting sympathetic stimulation, but, on the other hand, it induces inflammation and fibrosis in the kidney by upregulation of Ang II and reduction of Ang (1-7) [93, 94]. Therefore, to reduce intraglomerular pressure and proteinuria, NEP inhibitors are used in combination with an ACE or AT1R blocker to diminish the production or the effect of excess Ang II [95]. The use of an ACE inhibitor may be more effective than an AT1R blocker because of a shifting of Ang I to ACE2 or the NEP/Ang (1-7)/Mas axis.

Prolyl carboxypeptidase (PRCP) [92] and prolyl endopeptidase (PEP) [95] are lysosomal and cytosolic peptidase, respectively; they have been detected as the main enzymes in circulation (in white blood cells and plasma). Both enzymes are involved in the maturation and degradation of a variety of peptides and the conversion of Ang II to Ang (1-7), which may be considered as their protective role against Ang II-induced hypertension [92, 95, 96]. Previous studies demonstrated that PRCP participates in the processing of Ang II in human kidney extracts and cultured human glomerular endothelial cells [97]. However, more studies are needed to reveal their roles in kidney disease.

## 8 RAS therapeutic interventions used in diabetic nephropathy

DN, characterized by persistent proteinuria and a decline in GFR, is the leading cause of ESRD [1, 3]. In addition to controlling blood glucose, major therapeutic interventions include antihypertensive treatment and restriction of dietary proteins. Drug classes employed in DN include ACE inhibitors, angiotensin receptor blockers (ARB), beta-adrenergic blocking agents, calcium channel blockers, and diuretics [98]. The findings of clinical and animal studies suggest that ACE inhibitor users show a lower risk of ESRD compared with those on ARB [98, 99]. A meta-analysis of 119 randomized controlled trials (n = 64,768) concluded that ACE inhibitors provide more renal benefits and safety than ARBs [100]. ACE inhibitors, including benazepril, zofenopril, perindopril, trandolapril, captopril, enalapril, lisinopril, and ramipril lower blood pressure by causing relaxation of blood vessels via inhibition of ACE to produce Ang II and hydrolyze bradykinin [101]. In addition to being beneficial in cardiovascular diseases such as acute myocardial infarction and heart failure, they help reduce renal complications of DN by lowering blood pressure and strengthening perfusion in the glomerular artery [102]. ACE inhibitors are frequently the first choice of medication in DN [100]. All have common mechanisms, but according to the pharmacological reports, ACE inhibitors may differ in their affinity for tissue ACE [103]. One of the benefits of this treatment is the increase in Ang I due to the prevention of Ang II formation, which can be used to tread the alternative route of RAS by ACE2 to convert to Ang (1-7).

Ang (1-7) prevents the progression of nephropathy by ameliorating mesangial expansion, reducing oxidative stress and inflammation, and suppressing vascular smooth muscle cell proliferation and renal fibrosis. Ang (1-7) acts through downregulating TGF-β1/Smad signaling, blocking the activation of MAPKs and VEGF-mediated pathways, suppressing AT1R expression, and inducing ACE2 expression as a positive feedback mechanism via the Mas receptor [104]. ACE2 is not inhibited by ACE inhibitors and does not produce Ang II or metabolize bradykinin, thereby causing intrarenal vasodilation via stimulation of NO and vasoactive prostanoid production, which protects against a decrease in renal perfusion and GFR [105]. However, prior studies have demonstrated that patients treated with these inhibitors alone, even with effective doses, do not show suppression of Ang II formation [106]. As discussed above, Ang II may also be produced by ACE-independent Ang II formation pathways in human tissues that are insensitive to ACE inhibitors [92, 95]. Interestingly, ACE inhibitors increase bradykinin levels in tissues, which in turn cause mast cell degranulation, chymase release, and Ang II production. Therefore, in the presence of ACE inhibitors, Ang II can still be produced and maintained at a normal level [107]. For this reason, combination therapy is commonly used by some physicians to control blood pressure.

ARBs, including azilsartan, candesartan, eprosartan, irbesartan, losartan, olmesartan, telmisartan, and valsartan, are Ang II antagonists that bind to and inhibit AT1R [108]. Although the blockers suppress the AT1R pathway, in this condition, Ang II is free to stimulate further AT2R, which causes vasodilation and normalizes blood pressure. Both ARB and ACE inhibitors suppress RAS and slow the progression of DN, but increase blood potassium levels in diabetic patients with renal failure, which may be exacerbated by diabetic acidosis.

## 9 Conclusions

Patients with DN suffer from chronic hypertension because of decreased GFR and renal response through systemic RAS activation and elevated blood Ang II. In such conditions, in addition to precise control of blood sugar, the main treatment is to reduce RAS activity by ACE inhibitors or ARBs, use diuretics, and limit salt and protein intake to prevent hypertension and albuminuria.

In chronic hyperglycemia, the production of Ang II is also locally increased in the kidney, causing tissue damage, mainly through oxidative stress, inflammation, and the upregulation of certain growth factors and their receptors. There are alternative pathways in the kidney against local RAS which have been demonstrated to be protective in that they reduce tissue concentration of Ang II. Such autoregulatory pathways are disrupted in chronic kidney diseases. Thus, given the increased activity of local RAS, controlling blood pressure by inhibiting systemic RAS via ACE inhibitors or ARBs may not be sufficient to prevent the progression of renal failure. Further research with pharmaceutical and therapeutic approaches should be designed to strengthen the alternative pathways through upregulation of ACE2, neprilysin, and Ang (1-7), and interrupt other cell pathways related to local Ang II production.

## Abbreviations

ACE: Angiotensin-converting enzyme

ACEIs: ACE inhibitors

ADH: antidiuretic hormone

AGEs: advanced glycation end products

AMP: alanyl aminopeptidase

Ang II: angiotensin II

APA: aminopeptidase A

AT1R: angiotensin II receptor 1

AT2R: angiotensin II receptor 2

DN: diabetic nephropathy

ESRD: end-stage renal disease

Flk-1: fetal liver kinase 1

Flt-1: Fms-like tyrosine kinase 1

GBM: glomerular basement membrane

GFB: glomerular filtration barrier

GFR: glomerular filtration rate

GN: glomerulonephritis

HIF-1α: hypoxia-inducible factor α subunit

HO-1: heme oxygenase 1

ICAM-1: intercellular adhesion molecule 1

IFN: interferon

IL: interleukin

NEP: neprilysin

NO: nitric oxide

PAI: plasminogen activator inhibitors

RAS: renin-angiotensin system

PEP: prolyl endopeptidase

PHD: prolyl hydroxylase

Plt: platelet

PRCP: prolyl carboxypeptidase

RNS: reactive nitrogen species

ROS: reactive oxygen species

SMC: smooth muscle cell

TGF-β: transcription growth factor

VCAM-1: vascular cell adhesion molecule 1

VEGF: vascular endothelial growth factor

VEGFR: vascular endothelial growth factor receptor

## References

[1] Lim AK. Diabetic nephropathy-complications and treatment. Int J Nephrol Renovasc Dis 2014. 7:361-381. 10.2147/IJNRD.S40172 PMC4206379 25342915

[2] Alsaad KO, Herzenberg AM. Distinguishing diabetic nephropathy from other causes of glomerulosclerosis: an update. J Clin Pathol 2007. 60(1):18-26. 17213346 10.1136/jcp.2005.035592 PMC1860608

[3] Mason RM, Wahab NA. Extracellular matrix metabolism in diabetic nephropathy. J Am Soc Nephrol 2003. 14(5):1358-1373. 12707406 10.1097/01.asn.0000065640.77499.d7

[4] Arif E, Nihalani D. Glomerular filtration barrier assembly: an insight. Postdoc J 2013. 1(4):33-45. 27583259 PMC5003421

[5] Jefferson JA, Shankland SJ, Pichler RH. Proteinuria in diabetic kidney disease: a mechanistic viewpoint. Kidney Int 2008. 74(1):22-36. 18418356 10.1038/ki.2008.128

[6] Thomas MC, Forbes JM, Cooper ME. Advanced glycation end products and diabetic nephropathy. Am J Ther 2005. 12(6):562-572. 16280650 10.1097/01.mjt.0000178769.52610.69

[7] Donate-Correa J, Martín-Nunez E, Muros-de-Fuentes M, Mora-Fernandez C, Navarro-Gonzalez JF. Inflammatory cytokines in diabetic nephropathy. J Diabetes Res 2015. 2015:948417-948417. 25785280 10.1155/2015/948417 PMC4345080

[8] Mittal M, Siddiqui MR, Tran K, Reddy SP, Malik AB. Reactive oxygen species in inflammation and tissue injury. Antioxid Redox Signal 2014. 20(7):1126-1167. 23991888 10.1089/ars.2012.5149 PMC3929010

[9] Liu Y. Cellular and molecular mechanisms of renal fibrosis. Nature Rev Nephrol 2011. 7(12):684-696. 22009250 10.1038/nrneph.2011.149 PMC4520424

[10] Görlach A, Dimova EY, Petry A, Martinez-Ruiz A, HernansanzAgustin P, Rolo AP, Palmeira CM, Kietzmann T. Reactive oxygen species, nutrition, hypoxia and diseases: Problems solved? Redox Biol 2015. 6:372-385. 26339717 10.1016/j.redox.2015.08.016 PMC4565025

[11] Nayak BK, Shanmugasundaram K, Friedrichs WE, Cavaglierii RC, Patel M, Barnes J, Block K. HIF-1 mediates renal fibrosis in OVE26 type 1 diabetic mice. Diabetes 2016. 65(5):1387-1397. 26908870 10.2337/db15-0519 PMC4839204

[12] Masoud GN, Li W. HIF-1alpha pathway: role, regulation and intervention for cancer therapy. Acta Pharm Sin B 2015. 5(5):378-389. 10.1016/j.apsb.2015.05.007 PMC4629436 26579469

[13] Ramakrishnan S, Anand V, Roy S. Vascular endothelial growth factor signaling in hypoxia and inflammation. J Neuroimmune Pharmacol 2014. 9(2):142-160. 24610033 10.1007/s11481-014-9531-7 PMC4048289

[14] Tanaka S, Tanaka T, Nangaku M. Hypoxia and dysregulated angiogenesis in kidney disease. Kidney Dis (Basel) 2015. 1(1):80-89. 27536668 10.1159/000381515 PMC4934802

[15] Kim BS, Goligorsky MS. Role of VEGF in kidney development, microvascular maintenance and pathophysiology of renal disease. Kor J Intern Med 2003. 18(2):65-75. 10.3904/kjim.2003.18.2.65 PMC4531610 12872442

[16] Bartlett CS, Jeansson M, Quaggin SE. Vascular growth factors and glomerular disease. Ann Rev Physiol 2016. 78:437-461. 26863327 10.1146/annurev-physiol-021115-105412 PMC6450387

[17] Dulak J, Loboda A, Zagorska A, Jozkowicz A. Complex role of heme oxygenase-1 in angiogenesis. Antioxid Redox Signal 2004. 6(5):858-866. 15345146 10.1089/ars.2004.6.858

[18] Iglarz M, Touyz RM, Viel EC, Amiri F, Schiffrin EL. Involvement of oxidative stress in the profibrotic action of aldosterone. Interaction wtih the renin-angiotension system. Am J Hypertens 2004. 17(7):597-603. 15243979

[19] Zhao Q, Ishibashi M, Hiasa K, Tan C, Takeshita A, Egashira K. Essential role of vascular endothelial growth factor in angiotensin II-induced vascular inflammation and remodeling. Hypertension 2004. 44(3):264-270. 15262905 10.1161/01.HYP.0000138688.78906.6b

[20] Kang YS, Park YG, Kim BK, Han SY, Jee YH, Han KH, Lee MH, Song HK, Cha DR, Kang SW, Han DS. Angiotensin II stimulates the synthesis of vascular endothelial growth factor through the p38 mitogen activated protein kinase pathway in cultured mouse podocytes. J Mol Endocrinol 2006. 36(2):377-388. 16595708 10.1677/jme.1.02033

[21] Kim JH, Kim JH, Yu YS, Cho CS, Kim KW. Blockade of angiotensin II attenuates VEGF-mediated blood-retinal barrier breakdown in diabetic retinopathy. J Cereb Blood Flow Metab 2009. 29(3):621-628. 19107135 10.1038/jcbfm.2008.154

[22] Kitayama H, Maeshima Y, Takazawa Y, Yamamoto Y, Wu Y, Ichinose K, Hirokoshi K, Sugiyama H, Yamasaki Y, Makino H. Regulation of angiogenic factors in angiotensin II infusion model in association with tubulointerstitial injuries. Am J Hypertens 2006. 19(7):718-727. 16814127 10.1016/j.amjhyper.2005.09.022

[23] Tufro A, Veron D. VEGF and podocytes in diabetic nephropathy. Sem Nephrol 2012. 32(4):385-393. 10.1016/j.semnephrol.2012.06.010 PMC3438453 22958493

[24] Friis UG, Madsen K, Stubbe J, Hansen PB, Svenningsen P, Bie P, Skott O, Jensen BL. Regulation of renin secretion by renal juxtaglomerular cells. Pflugers Arch 2013. 465(1):25-37. 22733355 10.1007/s00424-012-1126-7

[25] Peti-Peterdi J, Harris RC. Macula densa sensing and signaling mechanisms of renin release. J Am Soc Nephrol 2010. 21(7):1093-1096. 20360309 10.1681/ASN.2009070759 PMC4577295

[26] Endo Y, Arima S, Yaoita H, Omata K, Tsunoda K, Takeuchi K, Abe K, Ito S. Function of angiotensin II type 2 receptor in the postglomerular efferent arteriole. Kidney Int Suppl 1997. 63:S205-207. 9407460

[27] Cravedi P, Remuzzi G. Pathophysiology of proteinuria and its value as an outcome measure in chronic kidney disease. Br J Clin Pharmacol 2013. 76(4):516-523. 23441592 10.1111/bcp.12104 PMC3791975

[28] Caldwell PR, Seegal BC, Hsu KC, Das M, Soffer RL. Angiotensin-converting enzyme: vascular endothelial localization. Science 1976. 191(4231):1050-1051. 175444 10.1126/science.175444

[29] Sparks MA, Crowley SD, Gurley SB, Mirotsou M, Coffman TM. Classical renin-angiotensin system in kidney physiology. Compr Physiol 2014. 4(3):1201-1228. 24944035 10.1002/cphy.c130040 PMC4137912

[30] Pontes RB, Girardi AC, Nishi EE, Campos RR, Bergamaschi CT. Crosstalk between the renal sympathetic nerve and intrarenal angiotensin II modulates proximal tubular sodium reabsorption. Exp Physiol 2015. 100(5):502-506. 25858030 10.1113/EP085075

[31] Giani JF, Janjulia T, Taylor B, Bernstein EA, Shah K, Shen XZ, McDonough AA, Bernstein KE, Gonzalez-Villalobos RA. Renal generation of angiotensin II and the pathogenesis of hypertension. Curr Hypertens Rep 2014. 16(9):477. 10.1007/s11906-014-0477-1 PMC4277187 25097114

[32] Campbell DJ. Clinical relevance of local renin angiotensin systems. Front Endocrinol 2014. 5:113. 10.3389/fendo.2014.00113 PMC4095645 25071727

[33] Madsen K, Marcussen N, Pedersen M, Kjaersgaard G, Facemire C, Coffman TM, Jensen BL. Angiotensin II promotes development of the renal microcirculation through AT1 receptors. J Am Soc Nephrol 2010. 21(3):448-459. 20056745 10.1681/ASN.2009010045 PMC2831863

[34] Dalal R, Bruss ZS, Sehdev JS. Physiology, renal blood flow and filtration. In: StatPearls. StatPearls Publishing, 2020. 29489242

[35] Ichikawa I, Brenner BM. Glomerular actions of angiotensin II. Am J Med 1984. 76(5b):43-49. 6203406 10.1016/0002-9343(84)90882-9

[36] Kalinowski L, Matys T, Chabielska E, Buczko W, Malinski T. Angiotensin II AT1 receptor antagonists inhibit platelet adhesion and aggregation by nitric oxide release. Hypertension 2002. 40(4):521-527. 12364357 10.1161/01.hyp.0000034745.98129.ec

[37] Kugler P. Aminopeptidase A is angiotensinase A-I. Quantitative histochemical studies in the kidney glomerulus. Histochemistry 1982. 74(2):229-245. 7174363 10.1007/BF00495833

[38] Fountain JH, Lappin SL. Physiology, Renin Angiotensin System. In: StatPearls. StatPearls Publishing StatPearls Publishing LLC. 2020. Sec. pp. 29261862

[39] Wright JW, Mizutani S, Harding JW. Focus on brain angiotensin III and aminopeptidase A in the control of hypertension. Int J. Hypertens 2012. 2012:124758-124758. 22792446 10.1155/2012/124758 PMC3389720

[40] Yacoub R, Campbell KN. Inhibition of RAS in diabetic nephropathy. Int J Nephrol Renovasc Dis 2015. 8:29-40. 25926752 10.2147/IJNRD.S37893 PMC4403683

[41] Peti-Peterdi J, Kang JJ, Toma I. Activation of the renal reninangiotensin system in diabetes--new concepts. Nephrol Dial Transplant 2008. 23(10):3047-3049. 18644796 10.1093/ndt/gfn377 PMC2720812

[42] Fathy SA, Mohamed MR, Ali MAM, El-Helaly AE, Alattar AT. Influence of IL-6, IL-10, IFN-gamma and TNF-alpha genetic variants on susceptibility to diabetic kidney disease in type 2 diabetes mellitus patients. Biomarkers 2019. 24(1):43-55. 30015512 10.1080/1354750X.2018.1501761

[43] Zhang J, Patel MB, Griffiths R, Mao A, Song Y-s, Karlovich NS, Sparks MA, Jin H, Wu M, Lin EE, Crowley SD. Tumor necrosis factor-α produced in the kidney contributes to angiotensin II-dependent hypertension. Hypertension 2014. 64(6):1275-1281. 25185128 10.1161/HYPERTENSIONAHA.114.03863 PMC4339088

[44] Liu X, Zhang H, Wang Q, Yu K, Wang R, Sun J. Blockade of vascular endothelial growth factor-A/receptor 2 exhibits a protective effect on angiotensin-II stimulated podocytes. Mol Med Rep 2015. 12(3):4340-4345. 26063200 10.3892/mmr.2015.3911

[45] Sanchez-Lopez E, Lopez AF, Esteban V, Yague S, Egido J, Ruiz-Ortega M, Alvarez-Arroyo MV. Angiotensin II regulates vascular endothelial growth factor via hypoxia-inducible factor-1alpha induction and redox mechanisms in the kidney. Antioxid Redox Signal 2005. 7(9-10):1275-1284. 16115033 10.1089/ars.2005.7.1275

[46] Timoshanko JR, Kitching AR, Iwakura Y, Holdsworth SR, Tipping PG. Leukocyte-derived interleukin-1beta interacts with renal interleukin-1 receptor I to promote renal tumor necrosis factor and glomerular injury in murine crescentic glomerulonephritis. Am J Pathol 2004. 164(6):1967-1977. 15161633 10.1016/s0002-9440(10)63757-1 PMC1615771

[47] Matavelli LC, Siragy HM. AT2 receptor activities and pathophysiological implications. J Cardiovasc Pharmacol 2015. 65(3):226-232. 25636068 10.1097/FJC.0000000000000208 PMC4355033

[48] Hunyady L, Catt KJ. Pleiotropic AT1 receptor signaling pathways mediating physiological and pathogenic actions of angiotensin II. Mol Endocrinol 2006. 20(5):953-970. 16141358 10.1210/me.2004-0536

[49] Berk BC. Angiotensin type 2 receptor (AT2R): a challenging twin. Sci STKE 2003. 2003(181):Pe16. 12734384 10.1126/stke.2003.181.pe16

[50] de Gasparo M, Levens NR. Pharmacology of angiotensin II receptors in the kidney. Kidney Int 1994. 46(6):1486-1491. 7699986 10.1038/ki.1994.426

[51] Kaschina E, Namsolleck P, Unger T. AT2 receptors in cardiovascular and renal diseases. Pharmacol Res 2017. 125(Pt A):39-47. 28694144 10.1016/j.phrs.2017.07.008

[52] Miyata N, Park F, Li XF, Cowley AW, Jr. Distribution of angiotensin AT1 and AT2 receptor subtypes in the rat kidney. Am J Physiol 1999. 277(3):F437-F446. 10484527 10.1152/ajprenal.1999.277.3.F437

[53] Carey RM, Padia SH. Role of angiotensin AT(2) receptors in natriuresis: Intrarenal mechanisms and therapeutic potential. Clin Exp Pharmacol Physiol 2013. 40(8):527-534. 23336117 10.1111/1440-1681.12059 PMC4011555

[54] Matavelli LC, Huang J, Siragy HM. Angiotensin AT_2_ receptor stimulation inhibits early renal inflammation in renovascular hypertension. Hypertension 2011. 57(2):308-313. 21189405 10.1161/HYPERTENSIONAHA.110.164202 PMC3060557

[55] Kemp BA, Howell NL, Keller SR, Gildea JJ, Padia SH, Carey RM. AT2 receptor activation prevents sodium retention and reduces blood pressure in angiotensin II-dependent hypertension. Circ Res 2016. 119(4):532-543. 27323774 10.1161/CIRCRESAHA.116.308384 PMC4975636

[56] Abadir PM, Carey RM, Siragy HM. Angiotensin AT2 receptors directly stimulate renal nitric oxide in bradykinin B2-receptor-null mice. Hypertension 2003. 42(4):600-604. 12953015 10.1161/01.HYP.0000090323.58122.5C

[57] Carey RM. Update on angiotensin AT2 receptors. Curr Opin Nephrol Hypertens 2017. 26(2):91-96. 27906747 10.1097/MNH.0000000000000304 PMC5813479

[58] Sumners C, de Kloet AD, Krause EG, Unger T, Steckelings UM. Angiotensin type 2 receptors: blood pressure regulation and end organ damage. Curr Opin Pharmacol 2015. 21:115-121. 25677800 10.1016/j.coph.2015.01.004 PMC4380821

[59] Matsubara H. Pathophysiological role of angiotensin II type 2 receptor in cardiovascular and renal diseases. Circ Res 1998. 83(12):1182-1191. 9851935 10.1161/01.res.83.12.1182

[60] Lavoie JL, Sigmund CD. Minireview: overview of the reninangiotensin system-an endocrine and paracrine system. Endocrinology 2003. 144(6):2179-2183. 12746271 10.1210/en.2003-0150

[61] Pugh D, Gallacher PJ, Dhaun N. Management of hypertension in chronic kidney disease. Drugs 2019. 79(4):365-379. 30758803 10.1007/s40265-019-1064-1 PMC6422950

[62] Peng H, Xing YF, Ye ZC, Li CM, Luo PL, Li M, Lou TQ. High glucose induces activation of the local renin-angiotensin system in glomerular endothelial cells. Mol Med Rep 2014. 9(2):450-456. 24337709 10.3892/mmr.2013.1855

[63] Peng H, Xing YF, Ye ZC, Li CM, Luo PL, Li M, Lou TQ. High glucose induces activation of the local reninangiotensin system in glomerular endothelial cells. Mol Med Rep 2014. 9(2):450-456. 24337709 10.3892/mmr.2013.1855

[64] Chen CM, Juan SH, Chou HC. Hyperglycemia activates the renin-angiotensin system and induces epithelial-mesenchymal transition in streptozotocin-induced diabetic kidneys. J Renin Angiotensin Aldosterone Syst 2018. 19(3):1470320318803009. 30264671 10.1177/1470320318803009 PMC6166313

[65] Uehara Y, Miura S, Yahiro E, Saku K. Non-ACE pathway-induced angiotensin II production. Curr Pharm Des 2013. 19(17):3054-3059. 23176219 10.2174/1381612811319170012

[66] Wasse H, Naqvi N, Husain A. Impact of Mast Cell Chymase on Renal Disease Progression. Curr Hypertens Rev 2012. 8(1):15-23. 23503162 10.2174/157340212800505007 PMC3596835

[67] Sharma R, Prasad V, McCarthy ET, Savin VJ, Dileepan KN, Stechschulte DJ, Lianos E, Wiegmann T, Sharma M. Chymase increases glomerular albumin permeability via protease-activated receptor-2. Mol Cell Biochem 2007. 297(1-2):161-169. 17102904 10.1007/s11010-006-9342-0

[68] Scandiuzzi L, Beghdadi W, Daugas E, Abrink M, Tiwari N, Brochetta C, Claver J, Arouche N, Zang X, Pretolani M, Monteiro RC. Mouse mast cell protease-4 deteriorates renal function by contributing to inflammation and fibrosis in immune complex-mediated glomerulonephritis. J Immunol 2010. 185(1):624-633. 20530261 10.4049/jimmunol.0902129 PMC2980698

[69] Jancso G, Jaberansari M, Gasz B, Szanto Z, Cserepes B, Röth E. Bradykinin and angiotensin-converting enzyme inhibition in cardioprotection. Exp Clin Cardiol 2004. 9(1):21-25. 19641692 PMC2716258

[70] Golias C, Charalabopoulos A, Stagikas D, Charalabopoulos K, Batistatou A. The kinin system--bradykinin: biological effects and clinical implications. Multiple role of the kinin system-bradykinin. Hippokratia 2007. 11(3):124-128. 19582206 PMC2658795

[71] Kakoki M, Smithies O. The kallikrein-kinin system in health and in diseases of the kidney. Kidney Int 2009. 75(10):1019-1030. 19190676 10.1038/ki.2008.647 PMC3733452

[72] Xu X, Tu L, Jiang W, Feng W, Zhao CX, Wang DW. Bradykinin prevents the apoptosis of NIT-1 cells induced by TNF-alpha via the PI3K/Akt and MAPK signaling pathways. Int J Mol Med 2012. 29(5):891-898. 22367460 10.3892/ijmm.2012.922

[73] Mayfield RK, Margolius HS, Levine JH, Wohltmann HJ, Loadholt CB, Colwell JA. Urinary kallikrein excretion in insulin-dependent diabetes mellitus and its relationship to glycemic control. J Clin Endocrinol Metab 1984. 59(2):278-286. 6564131 10.1210/jcem-59-2-278

[74] Jena P, Mohanty S, Mohanty T, Kallert S, Morgelin M, Lindstrøm T, Borregaard N, Stenger S, Sonawane A, Sørensen OE. Azurophil granule proteins constitute the major mycobactericidal proteins in human neutrophils and enhance the killing of mycobacteria in macrophages. PloS One 2012. 7(12):e50345-e50345. 23251364 10.1371/journal.pone.0050345 PMC3522671

[75] Rykl J, Thiemann J, Kurzawski S, Pohl T, Gobom J, Zidek W, Schluter H. Renal cathepsin G and angiotensin II generation. J Hypertens 2006. 24(9):1797-1807. 16915029 10.1097/01.hjh.0000242404.91332.be

[76] Korkmaz B, Horwitz MS, Jenne DE, Gauthier F. Neutrophil elastase, proteinase 3, and cathepsin G as therapeutic targets in human diseases. Pharmacol Rev 2010. 62(4):726-759. 21079042 10.1124/pr.110.002733 PMC2993259

[77] Ohlsson S, Falk R, Yang JJ, Ohlsson K, Segelmark M, Wieslander J. Increased expression of the secretory leukocyte proteinase inhibitor in Wegener’s granulomatosis. Clin Exp Immunol 2003. 131(1):190-196. 12519404 10.1046/j.1365-2249.2003.02024.x PMC1808594

[78] Becari C, Sivieri DO Jr, Santos CF, Moysss MK, Oliveira EB, Salgado MC. Role of elastase-2 as an angiotensin II-forming enzyme in rat carotid artery. J Cardiovasc Pharmacol 2005. 46(4):498-504. 16160604 10.1097/01.fjc.0000177982.68563.98

[79] Becari C, Silva MAB, Durand MT, Prado CM, Oliveira EB, Ribeiro MS, Salgado HC, Salgado MCO, Tostes RC. Elastase-2, an angiotensin II-generating enzyme, contributes to increased angiotensin II in resistance arteries of mice with myocardial infarction. Br J Pharmacol 2017. 174(10):1104-1115. 28222221 10.1111/bph.13755 PMC5406290

[80] Bader M. ACE2, angiotensin-(1-7), and Mas: the other side of the coin. Pflugers Arch 2013. 465(1):79-85. 23463883 10.1007/s00424-012-1120-0

[81] Clarke NE, Fisher MJ, Porter KE, Lambert DW, Turner AJ. Angiotensin converting enzyme (ACE) and ACE2 bind integrins and ACE2 regulates integrin signalling. PLoS One 2012. 7(4):e34747. 22523556 10.1371/journal.pone.0034747 PMC3327712

[82] Culver S, Li C, Siragy HM. Intrarenal angiotensin-converting enzyme: the old and the new. Curr Hypertens Rep 2017. 19(10):80-80. 28929450 10.1007/s11906-017-0778-2 PMC5913745

[83] Liu Z, Huang XR, Chen HY, Penninger JM, Lan HY. Loss of angiotensin-converting enzyme 2 enhances TGF-beta/Smad-mediated renal fibrosis and NF-kappaB-driven renal inflammation in a mouse model of obstructive nephropathy. Lab Invest 2012. 92(5):650-661. 22330342 10.1038/labinvest.2012.2

[84] Oudit GY, Liu GC, Zhong J, Basu R, Chow FL, Zhou J, Loibner H, Janzek E, Schuster M, Penninger JM, Herzenberg AM. Human recombinant ACE2 reduces the progression of diabetic nephropathy. Diabetes 2010. 59(2):529-538. 19934006 10.2337/db09-1218 PMC2809962

[85] Chappell MC. The angiotensin-(1-7) axis: formation and metabolism pathways. Angiotensin-(1-7): A Comprehensive Review, 2019, p. 1-26.

[86] Pinheiro SVB, Simoes E Silva AC. Angiotensin converting enzyme 2, Angiotensin-(1-7), and receptor MAS axis in the kidney. Int J Hypertens 2012. 2012:414128-414128. 22518283 10.1155/2012/414128 PMC3296191

[87] Alzayadneh EM, Chappell MC. Angiotensin-(1-7) abolishes AGE-induced cellular hypertrophy and myofibroblast transformation via inhibition of ERK1/2. Cell Signal 2014. 26(12):3027-3035. 25246357 10.1016/j.cellsig.2014.09.010 PMC4254268

[88] Zhang F, Ren X, Zhao M, Zhou B, Han Y. Angiotensin-(1-7) abrogates angiotensin II-induced proliferation, migration and inflammation in VSMCs through inactivation of ROS-mediated PI3K/Akt and MAPK/ERK signaling pathways. Sci Rep 2016. 6:34621-34621. 27687768 10.1038/srep34621 PMC5043354

[89] Zhang K, Meng X, Li D, Yang J, Kong J, Hao P, Guo T, Zhang M, Zhang Y, Zhang C. Angiotensin(1-7) attenuates the progression of streptozotocin-induced diabetic renal injury better than angiotensin receptor blockade. Kidney Int 2015. 87(2):359-369. 25075768 10.1038/ki.2014.274 PMC4317508

[90] Brar GS, Barrow BM, Watson M, Griesbach R, Choung E, Welch A, Ruzsicska B, Raleigh DP, Zraika S. Neprilysin is required for angiotensin-(1-7)’s ability to enhance insulin secretion via its proteolytic activity to generate angiotensin-(1-2). Diabetes 2017. 66(8):2201-2212. 28559246 10.2337/db16-1318 PMC5521860

[91] Ferrario CM, Chappell MC, Dean RH, Iyer SN. Novel angiotensin peptides regulate blood pressure, endothelial function, and natriuresis. J Am Soc Nephrol 1998. 9(9):1716-1722. 9727381 10.1681/ASN.V991716

[92] Grobe N, Weir NM, Leiva O, Ong FS, Bernstein KE, Schmaier AH, Morris M, Elased KM. Identification of prolyl carboxypeptidase as an alternative enzyme for processing of renal angiotensin II using mass spectrometry. Am J Physiol Cell Physiol 2013. 304(10):C945-C953. 23392115 10.1152/ajpcell.00346.2012 PMC3651643

[93] Iyer SN, Ferrario CM, Chappell MC. Angiotensin-(1-7) contributes to the antihypertensive effects of blockade of the reninangiotensin system. Hypertension 1998. 31(1 Pt 2):356-361. 9453328 10.1161/01.hyp.31.1.356

[94] Domenig O, Manzel A, Grobe N, Königshausen E, Kaltenecker CC, Kovarik JJ, Stegbauer J, Gurley SB, van Oyen D, Antlanger M, Bader M. Neprilysin is a mediator of alternative renin-angiotensin-system activation in the murine and human kidney. Sci Rep 2016. 6:33678-33678. 27649628 10.1038/srep33678 PMC5030486

[95] Seymour AA, Swerdel JN, Abboa-Offei B. Antihypertensive activity during inhibition of neutral endopeptidase and angiotensin converting enzyme. J Cardiovasc Pharmacol 1991. 17(3):456-465. 1711608 10.1097/00005344-199103000-00015

[96] Ardaillou R, Chansel D. Synthesis and effects of active fragments of angiotensin II. Kidney Int 1997. 52(6):1458-1468. 9407491 10.1038/ki.1997.476

[97] Velez JCQ, Ierardi JL, Bland AM, Morinelli TA, Arthur JM, Raymond JR, Janech MG. Enzymatic processing of angiotensin peptides by human glomerular endothelial cells. Am J Physiol Renal Physiol 2012. 302(12):F1583-F1594. 22461301 10.1152/ajprenal.00087.2012 PMC3378096

[98] Morgan TO, Anderson AI, MacInnis RJ. ACE inhibitors, beta-blockers, calcium blockers, and diuretics for the control of systolic hypertension. Am J Hypertens 2001. 14(3):241-247. 11281235 10.1016/s0895-7061(00)01266-8

[99] Molnar MZ, Kalantar-Zadeh K, Lott EH, Lu JL, Malakauskas SM, Ma JZ, Quarles DL, Kovesdy CP. Angiotensin-converting enzyme inhibitor, angiotensin receptor blocker use, and mortality in patients with chronic kidney disease. J Am Coll Cardiol 2014. 63(7):650-658. 24269363 10.1016/j.jacc.2013.10.050 PMC3944089

[100] Xie X, Liu Y, Perkovic V, Li X, Ninomiya T, Hou W, Zhao N, Liu L, Lv J, Zhang H, Wang H. Renin-angiotensin system inhibitors and kidney and cardiovascular outcomes in patients with CKD: a Bayesian network meta-analysis of randomized clinical trials. Am J Kidney Dis 2016. 67(5):728-741. 26597926 10.1053/j.ajkd.2015.10.011

[101] Hanif K, Bid HK, Konwar R. Reinventing the ACE inhibitors: some old and new implications of ACE inhibition. Hypertens Res 2010. 33(1):11-21. 19911001 10.1038/hr.2009.184

[102] Sun W, Zhang H, Guo J, Zhang X, Zhang L, Li C, Zhang L. Comparison of the efficacy and safety of different ACE inhibitors in patients with chronic heart failure: a PRISMA-compliant network meta-analysis. Medicine (Baltimore) 2016. 95(6):e2554-e2554. 26871774 10.1097/MD.0000000000002554 PMC4753869

[103] Dzau VJ, Bernstein K, Celermajer D, Cohen J, Dahlof B, Deanfield J, Diez J, Drexler H, Ferrari R, Van Gilst W, Hansson L. Pathophysiologic and therapeutic importance of tissue ACE: a consensus report. Cardiovasc Drugs Ther 2002. 16(2):149-160. 12090908 10.1023/a:1015709617405

[104] Patel VB, Zhong JC, Grant MB, Oudit GY. Role of the ACE2/ angiotensin 1-7 axis of the renin-angiotensin system in heart failure. Circ Res 2016. 118(8):1313-1326. 27081112 10.1161/CIRCRESAHA.116.307708 PMC4939482

[105] Mendoza-Torres E, Oyarzun A, Mondaca-Ruff D, Azocar A, Castro PF, Jalil JE, Chiong M, Lavandero S, Ocaranza MP. ACE2 and vasoactive peptides: novel players in cardiovascular/ renal remodeling and hypertension. Ther Adv Cardiovasc Dis 2015. 9(4):217-237. 26275770 10.1177/1753944715597623

[106] Ohnishi K, Murase M, Nakano D, Pelisch N, Hitomi H, Kobori H, Morimoto S, Mori H, Masaki T, Ohmori K, Kohno M. Angiotensin-converting enzyme inhibitor does not suppress renal angiotensin II levels in angiotensin I-infused rats. J Pharmacol Sci 2013. 122(2):103-108. 23698111 10.1254/jphs.13045fp PMC3786864

[107] Wei CC, Hase N, Inoue Y, Bradley EW, Yahiro E, Li M, Naqvi N, Powell PC, Shi K, Takahashi Y, Saku K. Mast cell chymase limits the cardiac efficacy of Ang I-converting enzyme inhibitor therapy in rodents. J Clin Invest 2010. 120(4):1229-1239. 20335663 10.1172/JCI39345 PMC2846039

[108] Miura SI, Karnik SS, Saku K. Review: angiotensin II type 1 receptor blockers: class effects versus molecular effects. J Renin Angiotensin Aldosterone Syst 2011. 12(1):1-7. 20603272 10.1177/1470320310370852 PMC3891529

